# Graph-based iterative Group Analysis enhances microarray interpretation

**DOI:** 10.1186/1471-2105-5-100

**Published:** 2004-07-23

**Authors:** Rainer Breitling, Anna Amtmann, Pawel Herzyk

**Affiliations:** 1 Plant Science Group, Institute of Biomedical and Life Sciences, University of Glasgow, Glasgow G12 8QQ, United Kingdom; 2 Bioinformatics Research Centre, Department of Computing Science, University of Glasgow, Glasgow G12 8QQ, United Kingdom; 3 Sir Henry Wellcome Functional Genomics Facility, Institute of Biomedical and Life Sciences, University of Glasgow, Glasgow G12 8QQ, United Kingdom

## Abstract

**Background:**

One of the most time-consuming tasks after performing a gene expression experiment is the biological interpretation of the results by identifying physiologically important associations between the differentially expressed genes. A large part of the relevant functional evidence can be represented in the form of graphs, e.g. metabolic and signaling pathways, protein interaction maps, shared GeneOntology annotations, or literature co-citation relations. Such graphs are easily constructed from available genome annotation data. The problem of biological interpretation can then be described as identifying the subgraphs showing the most significant patterns of gene expression. We applied a graph-based extension of our iterative Group Analysis (iGA) approach to obtain a statistically rigorous identification of the subgraphs of interest in any evidence graph.

**Results:**

We validated the Graph-based iterative Group Analysis (GiGA) by applying it to the classic yeast diauxic shift experiment of DeRisi et al., using GeneOntology and metabolic network information. GiGA reliably identified and summarized all the biological processes discussed in the original publication. Visualization of the detected subgraphs allowed the convenient exploration of the results. The method also identified several processes that were not presented in the original paper but are of obvious relevance to the yeast starvation response.

**Conclusions:**

GiGA provides a fast and flexible delimitation of the most interesting areas in a microarray experiment, and leads to a considerable speed-up and improvement of the interpretation process.

## Background

Microarray experiments can provide a comprehensive picture of gene expression levels in biological samples. In a typical application they compare expression of several thousand genes under two different conditions (e.g. healthy vs. diseased tissue, wild type vs. mutant animals, drug-treated vs. control cells), using a small number of replicate experiments. Various techniques have been developed to rank genes according to their expression changes, e.g. based on the t-statistic [1] or the strong non-parametric RankProducts [2]. The resulting list of genes can then be restricted to those genes that fulfill a certain statistical criterion, usually an arbitrarily chosen maximum accepted false discovery rate.

The main challenge to the biologist is contained in the next step of the analysis. It consists in identifying the biologically relevant expression changes, the "big picture" of the experiment. As microarray experiments tend to generate unexpected observations in areas outside the specialized expertise of the experimentalist, this can be quite difficult and time-consuming. A principled mechanism to identify the significant higher-level features of the experimental results would therefore be very useful.

The biological interpretation process consists to a large extent of finding evidence connecting certain genes that are differentially expressed. This evidence can consist, e.g., of joint participation in some physiological process, physical interaction at the protein level, reported co-expression in earlier microarray experiments, a shared functional annotation, etc. This kind of evidence can intuitively be represented as a graph, and this feature is regularly used to visualize biological data, in the form of metabolic or signaling pathways or protein interaction maps. The task can then be described as the identification of subgraphs that as a whole show a statistically significant expression change. This would allow the biologist to focus her analysis on the most promising areas, without prior bias, while at the same time presenting the relevant evidence underlying each association for critical evaluation.

## Results and Discussion

### The Algorithm

We have recently developed an approach, iterative Group Analysis (iGA) that identifies significantly changed functional classes of genes in a microarray experiment [3]. In contrast to similar approaches such as [4-8], the iGA method does not require a previous delimitation of a set of "differentially expressed genes", but uses an iterative calculation of p-values to determine the subset of class members that is most likely to be changed. Due to this feature, the iGA method is more sensitive in identifying functional classes that are slightly but consistently regulated, and works well on noisy data with small numbers of replicates, where the delimitation of gene lists can be overly restrictive.

**Figure 1:**
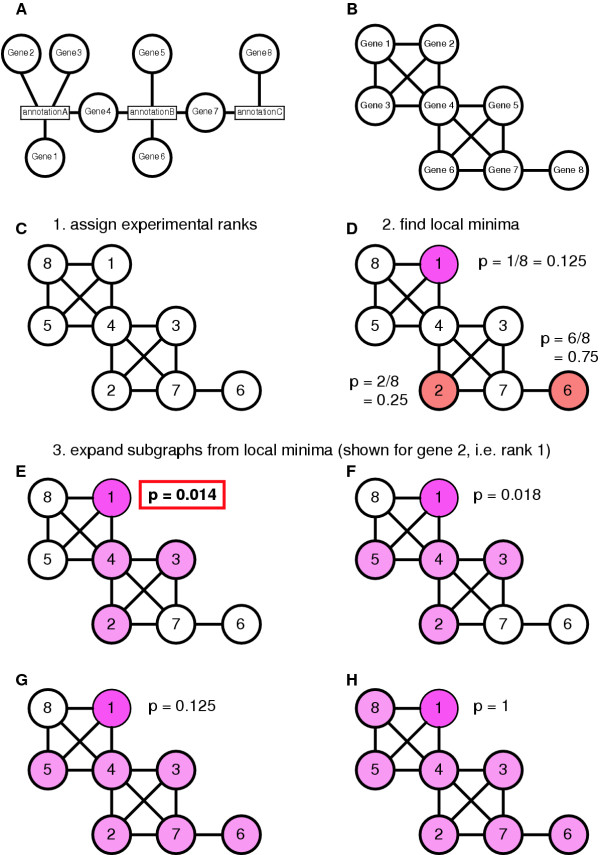
Principle of Graph-based Iterative Group Analysis. **A** Evidence network. Genes are associated with their annotation in the form of a bigraph (two types of nodes). **B** The same evidence represented as a simple network. Genes that share an annotation are connected. **C-H** Example of a GiGA analysis using fictitious microarray results. **C** Genes are assigned their ranks based on observed expression changes. **D** Local minima are found, i.e. genes that have no connection to genes with a better rank. **E-H** Iterative expansion of subgraphs from one of the local minima, gene 2 (rank 1). **E** The neighboring node with the smallest rank is included (gene 4, rank 4), which leads to the additional inclusion of genes 5 (rank 3) and 6 (rank 2). **F** Gene 3 (rank 5) is included). **G** Gene 7 (rank 7) is included, leading to the inclusion of gene 8 (rank 6). **H** The last gene reachable from this local minimum, gene 1 (rank 8), is included and the process terminates. For each of the subgraphs a p-value can be calculated (see text) and the subgraph with the smallest p-value is declared the "regulated neighborhood" of the local minimum. In the example, genes 2, 4, 5, and 6 form a regulated neighborhood (p = 0.014). The graph expansion process would then be repeated for the remaining two local minima.

Here we extend this approach to the analysis of "evidence graphs", which offers much larger flexibility of the annotations that can be used and allows substantially improved visualization. Evidence graphs can be represented as bigraphs with two types of nodes, one for genes and one for the associated "evidence" (Fig. 1A). For evaluation purposes we focused on two types of networks, one where the evidence consists of GeneOntology annotations (GO network) and one where the evidence comprises enzyme substrates (metabolic network). The construction of these networks from gene annotation files is fast and simple, but more unconventional networks are also straightforward (e.g. regulatory networks inferred from previous gene expression experiments, or "literature networks" based on co-occurrence of genes in publications). Before the calculation the bigraph is converted to a simple graph, eliminating the evidence nodes and introducing pairwise edges between all nodes that were connected to a common evidence node (Fig. 1B).

In addition to the graph, a complete list of genes sorted by differential expression is provided. All nodes without corresponding expression data are eliminated from the network. In the first step of the analysis, each gene node is assigned its rank in the list of genes, such that the node for the most strongly changed gene is labeled "1" (Fig. 1C), the second most changed gene is labeled "2", etc. Then, local minima are identified in the graph, i.e. those nodes that have a lower rank than all their direct neighbors in the graph (Fig. 1D). In the next step, subgraphs are iteratively extended from each of those local minima by including the neighboring node with the next highest rank (*m*) and, if present, all adjacent nodes of ranks equal or smaller than *m*. Hence, at each step of the extension process the newly extended subgraph is not adjacent to any outside node with a rank lower than *m* (Fig. 1E,1F,1G,1H). At each extension step we thus obtain a subgraph with *n* members with a maximum rank *m* and can calculate the *p*-value for observing all *n* of *n* genes at rank *m* or better in a list of *N* total genes in the graph, 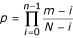, which follows easily from inserting these values in the cumulative hypergeometric equation, which is also used for iGA [3].

The extension process is continued until all nodes reachable from the local minimum are included or the subgraph reaches an arbitrary maximum size. After extending the subgraphs, for each local minimum the subgraph yielding the smallest p-value is selected as its "regulated neighborhood" and all local minima are sorted by increasing p-value of these regulated neighborhoods. The subgraphs at the top positions of the resulting list should contain the most relevant regions of the total evidence network.

### Comparison with Previous Approaches

The method devised by Ideker et al. [9] for the determination of signaling and regulation circuits from a combination of protein interaction information and expression data could easily be extended to cover the more general case of microarray interpretation addressed by GiGA. This would only require the extension of "interaction" to include, e.g., participation in a shared cellular process or shared functional annotation. Their approach uses aggregate z-scores to evaluate the quality of each subgraph. This requires a relatively complex parameter estimation procedure followed by simulated annealing. In contrast, the rank-transform of the data that is the basis of GiGA allows non-parametric p-value calculations and is thus much faster (and computationally less demanding) than the method described by Ideker et al. A disadvantage of both methods is that they both do not guarantee to find the optimally scoring subgraph.

The recently released commercial Pathway Analysis software from Ingenuity Systems  seems to be based on a similar concept as GiGA, i.e. the determination of regulated subgraphs in annotation networks. However, it just classifies genes as changed ("focus genes") or unchanged based on an arbitrary selected significance cut-off and thus discards most of the relative-change information (gene ranks) used by GiGA . Therefore, this method is difficult to apply to very noisy or unreplicated experiments where a reliable delimitation of the "changed" genes becomes impossible.

### Experimental Case Study

To validate the GiGA approach, we used the yeast diauxic shift experiment by DeRisi et al. [10]. In this classical study the authors examined the response of yeast cells to glucose depletion in the growth medium. As the biology of this process is extremely well understood and the functional annotation of the yeast genome is very comprehensive, we could use this dataset to test the ability (and reliability) of GiGA to identify the relevant subgraphs of interest.

In their original publication, DeRisi et al. highlight the following changes during starvation: Rechanneling of metabolites into the tricarboxylic acid (TCA) and glyoxylate cycle, increase in aerobic respiration (cytochrome c oxidase and reductase), gluconeogenesis, and carbohydrate storage (glycogen and trehalose biosynthesis). In contrast, 95% of ribosomal proteins, as well as tRNA synthetases and translation elongation/initiation factors were strongly down-regulated. Twenty hours after the initial inoculation of the sample about 20% of all genes showed at least a two-fold change in expression.

Table 1 and 2 show the result of an iterative Group Analysis of these expression data. Seven time points after inoculation were examined. Significant expression changes become apparent at 13.5 hours. It can be seen that all the processes identified manually by DeRisi et al. are already apparent at this level of analysis. In addition, iGA highlights the up-regulation of sugar transporters at the early stages of starvation, obviously a desperate attempt by the cells to take up the last remaining sugars from the medium. It also highlights the induction of heat shock proteins and the repression of ribosome biosynthesis processes in the nucleolus, which were not discussed in the original paper. What is missing in these lists, however, is the automatic identification of the interrelationships between the identified processes, which would be particularly informative in more realistic applications with a less well understood biology.

**Figure 2:**
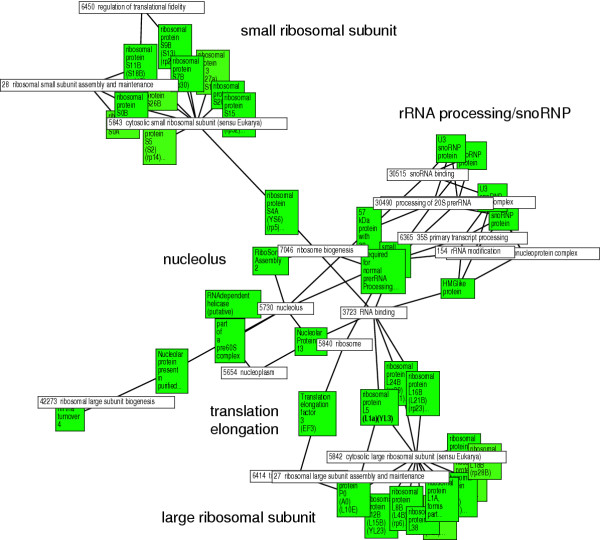
Visualization of the most significant "down-regulated neighborhood" identified by GiGA using a GeneOntology-based network. The expression data are taken from the 20.5 h timepoint of the yeast diauxic shift (DeRisi et al., 1997). The layout was generated from the output of GiGA by the free software aiSee  using a force-directed algorithm with default parameters. The same software can also be used for the versatile real-time navigation of the network. Colored boxes show the regulated genes (darker shading indicates stronger regulation), white boxes show the evidence linking the genes (in this case GeneOntology numbers and terms). Several important components of this regulatory neighborhood are indicated (small and large ribosomal subunit proteins, rRNA processing/snRNP, nucleolar proteins, translation elongation factors). These components were also identified in the original publication after manual analysis. GiGA finds them automatically, and also detects the – biologically obvious – connections between them. As all the evidence is included in the same picture, the biologist can then use her expertise to assess the relevance of each link, without having to make the connections *ad hoc* by tedious literature studies.

**Figure 3:**
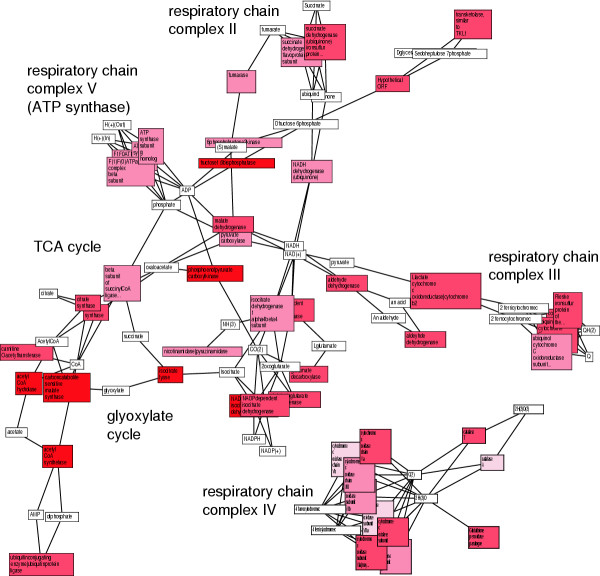
Visualization of the two most significant "up-regulated neighborhoods" identified by GiGA using a metabolic network derived from Swissprot annotations. The expression data are taken from the 20.5 h timepoint of the yeast diauxic shift (DeRisi et al., 1997). The layout was generated as in Fig 2. Colored boxes show the regulated genes (darker shading indicates stronger regulation), white boxes show the substrates that are in common between genes. Important components of this regulatory neighborhood are indicated (TCA cycle and glyoxylate cycle enzymes, and the various respiratory chain complexes). Here it can be seen that GiGA not only detects protein complexes (such as ribosomes or the respiratory chain complexes), but also "linear" metabolic pathways such as TCA cycle and glyoxylate cycle (and potentially signal transduction pathways or regulatory cascades etc.). Almost all the enzymes discussed by DeRisi et al. (1997) are included in these two subgraphs, plus the relevant enzymatic information necessary to assess the relevance of each link, without the danger of missing some genes (unless the annotation is incomplete).

This connectivity between genes and functional classes is provided by GiGA. Table 3 summarizes the results for the 20.5 hour time point, using two different networks, one for GeneOntology classes, and one for enzyme substrates, extracted from the SwissProt catalytic activity descriptors of yeast proteins. For both up- and down-regulated genes, the most significant subgraph is widely separated from the next best one, contains the largest number of genes, and comprises almost all the processes detected by iGA and the original authors. (We here restricted the size of subgraphs to a maximum of 40 genes to keep navigation of the results simple.) Figures 2 and 3 show the automatically generated visualization of the corresponding most significant subgraphs and the associated annotation. It is obvious how GiGA highlights the functional connections between different enzymes. In Fig. 2, the association between small and large ribosomal subunits, nucleolar rRNA processing and translational elongation is faithfully reconstructed. In Fig. 3, which uses the enzyme substrate network (which we considered to be more challenging for the algorithm), the interplay between the TCA cycle, the overlapping glyoxylate cycle, and all the relevant protein complexes of the respiratory chain are readily apparent. The agreement with the manual interpretation by DeRisi et al. extends down to the single gene level, while at the same time adding additional, obviously relevant connections, e.g. from the TCA cycle to the ATP synthase complex. Fig. 3 also shows the second best subgraph, which contains the cytochrome c oxidase subunits together with two catalase genes that may be involved in the detoxification of the hydrogen peroxide generated by the respiratory burst induced by starvation.

The performance of GiGA (as well as iGA) is best appreciated when compared to the results of an extensive expert interpretation of the same data. Table 1 to 3, and Fig. 2 and 3 show how both techniques succeed in detecting and condensing exactly the genes and processes that were considered relevant by the expert biologists when first interpreting the same data [10]. GiGA effectively summarizes the original publication in three subgraph pictures (Fig. 2 and 3). This is even more astonishing when considering that these results are achieved for each single time-point separately (see, e.g., Tab. 1 and 2). This reveals the stability of the approach towards the measurement variance inherent in any unreplicated microarray experiment.

It is important to be aware that each of the highlighted subgraphs has to be carefully evaluated for its biological relevance. On the one hand, the sheer number of possible subgraphs in an evidence network creates a major multiple-testing problem, which means that some of the detected associations may be due to chance. Random permutations of the expression data – which can be generated by the GiGA software – can give an idea of the expected false-discovery rate. On the other hand, functional annotations are at present notoriously unreliable and spurious edges may affect the details of the results. Also, not all genes within a detected subgraph will necessarily show a strong expression change, because sometimes less strongly affected genes may connect those genes that do change. Such a relation is for example expected for many transcription factors and their targets [9]. Nonetheless, GiGA is able to direct the user to the most interesting areas in the evidence network.

The GiGA method is not restricted to use with exhaustively annotated genomes. It can work on a wide variety of "evidence" to build the necessary network, including hypothetical predicted functions or associations. It is even possible to apply GiGA to metabolomics results, which are characterized by the absence of any significant amount of annotation – usually only exact molecular masses and their differential abundance are known. In that case, an evidence network can be built from the measured masses themselves, linking compounds *m*_1_ and *m*_2_ whenever their mass difference (Δ*m* = |*m*_1_ - *m*_2_|) can be explained by a common biochemical transformation (e.g. dehydrogenation: Δ*m* = 2* *mass*(*hydrogen*)). The set of relevant transformations can easily be collected from any biochemistry textbook. In addition, one can introduce edges for condensation reactions between observed masses, i.e. if m_1_ + m_2_ = m_3_ + mass(H_2_O) then edges between m_1_ and m_3_, and m_2_ and m_3_ are added to the evidence network. We are currently developing the application of GiGA to this kind of data in the Sir Henry Wellcome Functional Genomics Facility at the University of Glasgow ; data not shown).

## Conclusions

The present analysis of a biologically well-understood test case demonstrates the reliable performance of GiGA. The method automatically identifies all relevant physiological processes, puts them into context, summarizes them in an intuitive format, and associates them with the underlying evidence (Fig. 2 and 3). It can be applied to experiments with very small numbers of replicates (a single time point in the diauxic shift test case) and can be used with any available functional annotation, including protein interaction networks, co-expression data or literature mining results, as well as in areas beyond microarray analysis. For visualization, we have used the graph-layout software aiSee, but output files suitable for a variety of graphical tools can easily be generated by slight modifications in the implementation. GiGA can be used as a stand-alone tool, but we expect that it will be most useful when integrated into existing microarray analysis software, and for that reason the GiGA algorithm is freely available without restrictions.

## Methods

Yeast gene expression data for the diauxic shift experiment were obtained from the Stanford Microarray Database . GeneOntology annotations  were obtained from Affymetrix . The enzyme substrate networks were built based on information contained in the annotation of the yeast proteome in the SwissProt database . The GiGA algorithm has been implemented as a Perl script and compiled as a Windows command line executable. These files are available (together with a manual and example files) as Additional files 1 to 8.

## Authors' contributions

RB devised and implemented the GiGA technique and drafted the manuscript. AA and PH supervised the project. All authors read and approved the final manuscript.

## Supplementary Material

Additional File 1 GiGA program. For use from the Windows command line. Click here for file

Additional File 2 GiGA source code. Click here for file

Additional File 3 GiGA manual. Describes the use of GiGA applied to the example data (Additional files 4 to 6). Click here for file

Additional File 4 Gene expression data. Sorted list of genes, based on expression during the yeast diauxic shift. Click here for file

Additional File 5 Evidence network. List of gene pairs connecting genes whenever their gene products are enzymes that share a common substrate. Based on annotation derived from SwissProt. Click here for file

Additional File 6 Genenames file. Contains descriptive names of the yeast genes contained in Additional file 4. Click here for file

Additional File 7 Example output in text format. List of significantly affected subgraphs detected in the experimental data (Additional file 4) using GiGA with default settings. Click here for file

Additional File 8 Example output in graph-description language format. Contains the same results as Additional file 7, but in a format that can be visualized and explored using graph-layout software, e.g. aiSee, which is freely available for academics at . Click here for file

## Figures and Tables

**Table 1:** Iterative Group Analysis of gene expression during the yeast diauxic shift. Up-regulated groups.

**0 h**	**9.5 h**	**11.5 h**	**13.5 h**	**15.5 h**	**18.5 h**	**20.5 h**
		6144 – purine base metabolism	**6099 – tricarboxylic acid cycle**	**6099 – tricarboxylic acid cycle**	3773 – heat shock protein activity	**6099 – tricarboxylic acid cycle**
		9277 – cell wall (sensu Fungi)	3773 – heat shock protein activity	**5749 – respiratory chain complex II (sensu Eukarya)**	**6099 – tricarboxylic acid cycle**	3773 – heat shock protein activity
		297 – spermine transporter activity	6950 – response to stress	**6121 – oxidative phosphorylation, succinate to ubiquinone**	**5977 – glycogen metabolism**	**5749 – respiratory chain complex II (sensu Eukarya)**
		15846 – polyamine transport	297 – spermine transporter activity	**8177 – succinate dehydrogenase (ubiquinone) activity**	6950 – response to stress	**6121 – oxidative phosphorylation, succinate to ubiquinone**
			**4373 – glycogen (starch) synthase activity**	3773 – heat shock protein activity	**4373 – glycogen (starch) synthase activity**	**8177 – succinate dehydrogenase (ubiquinone) activity**
			15846 – polyamine transport	**4373 – glycogen (starch) synthase activity**	**4129 – cytochrome c oxidase activity**	6537 – glutamate biosynthesis
			5353 – fructose transporter activity	7039 – vacuolar protein catabolism	**5751 – respiratory chain complex IV (sensu Eukarya)**	**6097 – glyoxylate cycle**
			15578 – mannose transporter activity	6950 – response to stress	**5749 – respiratory chain complex II (sensu Eukarya)**	**5750 – respiratory chain complex III (sensu Eukarya)**
			7039 – vacuolar protein catabolism	**4129 – cytochrome c oxidase activity**	**6121 – oxidative phosphorylation, succinate to ubiquinone**	**9060 – aerobic respiration**
			8645 – hexose transport	**5751 – respiratory chain complex IV (sensu Eukarya)**	**8177 – succinate dehydrogenase (ubiquinone) activity**	**4129 – cytochrome c oxidase activity**
			4396 – hexokinase activity	4396 – hexokinase activity	30162 – regulation of proteolysis and peptidolysis	**5751 – respiratory chain complex IV (sensu Eukarya)**
			5215 – transporter activity	297 – spermine transporter activity	4364 – glutathione transferase activity	16491 – oxidoreductase activity
			**5977 – glycogen metabolism**			**6101 – citrate metabolism**

For this analysis, genes were assigned to groups based on GeneOntology annotations  obtained from Affymetrix . All groups that are changed with a minimal p-value smaller than 1/ [number of annotated genes] (1/4087 = 2.4E-4) are shown, sorted by significance. Numbers and names are the standardized GeneOntology identifiers. Groups shown in bold were also reported as changed in the original publication (DeRisi et al., 1997) Up-regulated groups

**Table 2:** Iterative Group Analysis of gene expression during the yeast diauxic shift.

**0 h**	**9.5 h**	**11.5 h**	**13.5 h**	**15.5 h**	**18.5 h**	**20.5 h**
7152 – spore wall assembly (sensu Saccharomyces)			**5730 – nucleolus**		**30490 – processing of 20S pre-rRNA**	**5843 – cytosolic small ribosomal subunit (sensu Eukarya)**
			**30490 – processing of 20S pre-rRNA**		**5732 – small nucleolar ribonucleoprotein complex**	**5842 – cytosolic large ribosomal subunit (sensu Eukarya)**
			**7046 – ribosome biogenesis**		**5730 – nucleolus**	**30490 – processing of 20S pre-rRNA**
			**30515 – snoRNA binding**		**42273 – ribosomal large subunit biogenesis**	**5730 – nucleolus**
					**5842 – cytosolic large ribosomal subunit (sensu Eukarya)**	**6414 – translational elongation**
					**30515 – snoRNA binding**	**5732 – small nucleolar ribonucleoprotein complex**
					3938 – IMP dehydrogenase activity	**27 – ribosomal large subunit assembly and maintenance**
					6183 – GTP biosynthesis	**42273 – ribosomal large subunit biogenesis**
					**154 – rRNA modification**	**30515 – snoRNA binding**
						**3723 – RNA binding**
						**154 – rRNA modification**

Down-regulated groups. See Table 1 for details. Down-regulated groups.

**Table 3:** Graph-based iterative Group Analysis of gene expression during the yeast diauxic shift.

**Down-regulated genes using GeneOntology-based network**					
**anchor locus**	**group description**	**minimal p-value**	**E-value**	**N**	**max. rank**

YHL015W	***ribosomal proteins and rRNA processing***	5.87E-86	<0.01	39	48
YMR217W	amino acid and nucleotide biosynthesis	3.38E-13	2.7	9	172
YDR144C	cell wall biogenesis	4.06E-08	4.5	6	242
YNL065W	membrane transporter	4.02E-05	9.3	3	141
YLR062C	bud site selection	6.41E-05	9.9	4	367
YGL225W	protein glycosylation in Golgi	1.12E-04	10.8	4	422
YPR074C	pentose phosphate pathway	1.44E-04	11.2	4	449
**total genes measured in network: 4087.**					
**Down-regulated genes using metabolic network**					

**anchor locus**	**group description**	**minimal p-value**	**E-value**	**N**	**max. rank**

YNL141W	***nucleotide and amino acid biosynthesis, tRNA synthetases***	4.67E-59	<0.01	39	45
YOR224C	RNA polymerases	2.59E-13	1.1	23	219
**total genes measured in network: 744.**					
**Up-regulated genes using GeneOntology-based network**					

**anchor locus**	**group description**	**minimal p-value**	**E-value**	**N**	**max. rank**

YER065C	***TCA and glyoxylate cycle, respiratory chain***	8.57E-77	<0.01	39	66
YKL217W	membrane transporters (sugar, amino acids)	1.76E-15	2.3	8	62
YAL017W	protein kinases	1.07E-07	4.8	6	284
YBL043W	cell wall biogenesis	3.81E-07	5.4	4	103
YGR248W	carbohydrate metabolism	5.66E-07	5.5	5	232
YEL011W	***glycogen metabolism***	1.01E-06	5.8	3	42
YER037W	protein phosphatases	1.07E-06	5.8	8	736
YJL137C	***glycogen biosynthesis***	7.46E-06	7.3	4	215
YDL085W	disulfide oxidoreductases	1.05E-05	7.6	4	234
YNL173C	mating signal transduction	1.65E-05	8.2	4	262
YNL134C	alcohol dehydrogenase	1.34E-04	11.1	3	210
YBL038W	***mitochondrial large ribosomal subunit***	1.99E-04	11.9	4	487
**total genes measured in network: 4087.**					
**Up-regulated genes using metabolic network**					

**anchor locus**	**group description**	**minimal p-value**	**E-value**	**N**	**max. rank**

YER065C	***TCA and glyoxylate cycle, respiratory chain***	4.96E-53	0.11	39	54
YGR088W	***cytochrome c oxidase***	3.09E-10	1.2	11	106
YFR015C	***glycogen synthases***	2.08E-04	3.6	3	45
YJR073C	methyltransferases	3.85E-04	4.0	5	156
YDR001C	***trehalases***	5.01E-04	4.2	3	60
YCR014C	DNA and RNA polymerases	5.44E-04	4.2	17	481
YIR038C	glyoxalases	8.64E-04	4.5	5	183
**total genes measured in network: 744.**					

The evidence network was constructed either from GeneOntology information (nodes are connected if they share a GeneOntology annotation) or from enzyme activity information obtained from Swissprot . In the latter case, genes are connected if their encoded proteins convert the same substrate (as product or educt, i.e. the direction of the reaction is not taken into account here). This type of network is much smaller (only 744 genes), as only genes coding for enzymes are included. All groups that are changed with a minimal p-value smaller than 1/[number of annotated genes] are shown, sorted by significance. The corresponding E-value as estimated by the analysis of 100 random permutations of the data is also shown. The employed threshold for inclusion in the table is very generous and does not guarantee that all subgraphs shown are statistically significant. The local minimum anchoring each regulated neighborhood is indicated by its genetic locus name (for overlapping neighborhoods, only the best-ranking minimum is shown). Descriptive group names were added manually. Groups that correspond to processes discussed in the original paper are highlighted in italics. It can be seen that the highest ranking group in each case is the largest and contains the central biological processes detected by DeRisi et al. (1997) and by iGA (see Table 1 and 2). N, number of genes in each subgraph.
