# Supplementary material for: Graph-based iterative Group Analysis enhances microarray interpretation
Source: BMC Bioinformatics. 2004 Jul 23;5:100. doi: 10.1186/1471-2105-5-100 (PMC509016; doi:10.1186/1471-2105-5-100)
Supplement: Additional File 3 — GiGA manual. Describes the use of GiGA applied to the example data (Additional files 4 to 6). [file 1471-2105-5-100-S3.pdf]

# Instruction for the use of Graph-based Iterative Group Analysis

If you want to test Graph-based Iterative Group Analysis yourself, you can either use the Perl script (`GiGA.pl`) or the Microsoft Windows executable (`GiGA.exe`), which are both included in the supplementary material. The instructions below refer to the Windows version, but the Perl script uses the same syntax.

## Installing GiGA

To install GiGA on your computer just unpack all files into a single new directory (e.g. `C:\"My Documents"\GiGA`).

## Starting GiGA

The current GiGA implementation is a command line program. If you want to start it in Windows, you first have to open the Command prompt. This is done by selecting Accessories > Command Prompt in the Start menu.

To start GiGA change to the GiGA directory:

```
C:\> cd "My Documents"\GiGA
```

Then call the program:

```
C:\My Documents\GiGA\> GiGA.exe
```

This will print a summary of the options available.

## Example analysis

You may then want to try an analysis of the example files provided, which is taken from the diauxic shift experiment described in the manuscript, with an evidence network based on shared enzyme substrates:

```
C:\My Documents\GiGA\> GiGA.exe –Iexperimental_data.txt
-Nevidence_network.txt -Ggenenames.txt -Ftxt
```

The option -I indicates the input file which contains the sorted list of genes to be analyzed, -N specifies the evidence network file prepared from gene annotations, -G the file that contains the descriptive gene names associated with the gene identifiers.

After a few moments (less than 30 seconds on a 2 GHz computer) this should print the following analysis:

```
YER065C isocitrate lyase       1.03717722619693e-024  37
-1-     YER065C isocitrate lyase       1       2
-2-     YKR097W phosphoenolpyruvate carboxylkinase    2       3
-3-     YAL054C acetyl CoA synthetase  4       7
-4-     YBL015W acetyl CoA hydrolase   5       13
-5-     YLR174W NADP-dependent isocitrate dehydrogenase      6       14
-6-     YNL117W carbon-catabolite sensitive malate synthase  7       15
-7-     YNR001C citrate synthase       8       18
-8-     YMR250W glutamate decarboxylase       9       19
-9-     YIL125W alpha-ketoglutarate dehydrogenase     10      26
-10-    YKL085W malate dehydrogenase   19      58
-11-    YOR374W aldehyde dehydrogenase 27      72
```

-12- YEL012W ubiquitin-conjugating enzyme|ubiquitin-protein ligase 28 78
-13- YCR005C citrate synthase 31 90
-14- YMR170C aldeyhde dehydrogenase 33 95
-15- YNL009W NADP-dependent isocitrate dehydrogenase 35 106
-16- YDL215C NAD-dependent glutamate dehydrogenase 36 110
-17- YML042W carnitine O-acetyltransferase 37 113
YGR088W catalase T 3.09133753959947e-010 106
-1- YGR088W catalase T 12 29
-2- YKL026C Glutathione peroxidase paralogue 14 41
-3- YHR051W cytochrome c oxidase subunit 23 66
-4- YGL191W cytochrome c oxidase subunit VIa|may specifically interact with ATP 30
89
-5- YNL052W cytochrome c oxidase chain Va 34 98
-6- YGL187C cytochrome c oxidase subunit IV 38 114
-7- YLR395C cytochrome c oxidase chain VIII 55 195
-8- YLR038C cytochrome c oxidase subunit VIb 71 285
-9- YDL067C cytochrome c oxidase subunit VIIa 96 380
-10- YIL111W cytochrome c oxidase chain Vb 99 395
-11- YDR256C catalase A 106 430
YML054C L-lactate cytochrome c oxidoreductase|cytochrome b2 5.79426946267908e-007 44
-1- YML054C L-lactate cytochrome c oxidoreductase|cytochrome b2 11 28
-2- YDR529C ubiquinol cytochrome C oxidoreductase subunit 7 (14 kDa) 21 63
-3- YBL045C coenzyme QH2 cytochrome c reductase 44 kDa core protein subunit 24
67
-4- YEL024W Rieske iron-sulfur protein of the mitochondrial cytochrome bc1 complex
32 92
-5- YFR033C ubiquinol cytochrome C oxidoreductase subunit 6 (17 kDa) 44 140
YLR377C fructose-1,6-bisphosphatase 3.80328564091156e-005 26
-1- YLR377C fructose-1,6-bisphosphatase 3 4
-2- YGR043C Hypothetical ORF 15 45
-3- YBR117C transketolase, similar to TKL1 26 70
YJL045W Similar to SDH1 0.000155934711277374 41
-1- YJL045W Similar to SDH1 20 60
-2- YLL041C succinate dehydrogenase (ubiquinone) iron-sulfur protein subunit 22
64
-3- YKL148C succinate dehydrogenase flavoprotein subunit 41 126
YFR015C glycogen synthase (UDP-glucose-starch glucosyltransferase)
0.000207571627863596 45
-1- YFR015C glycogen synthase (UDP-glucose-starch glucosyltransferase) 13 36
-2- YLR258W glycogen synthase (UDP-glucose-starch glucosyltransferase) 18 57
-3- YML100W similar to TPS3 gene product|trehalose-6-phosphate synthase/phosphatase
complex 123 kDa regulatory subunit 45 144
YJR073C unsaturated phospholipid N-methyltransferase 0.000385031670742329 156
-1- YJR073C unsaturated phospholipid N-methyltransferase 53 188
-2- YGL192W methyltransferase 89 346
-3- YDL033C Hypothetical ORF 131 765
-4- YOL096C 3,4-dihydroxy-5-hexaprenylbenzoate methyltransferase 151 946
-5- YML110C C-methyltransferase (putative) 156 970
YDR001C neutral trehalase 0.000500570902430744 60
-1- YDR001C neutral trehalase 49 164
-2- YBR001C neutral trehalase 58 211
-3- YPR026W acid trehalase 60 227
YCR014C DNA polymerase IV 0.000543856186478769 481
-1- YCR014C DNA polymerase IV 199 1460
-2- YBR278W DNA polymerase II C and C' subunits 220 1732
-3- YOR210W RNA polymerase II core subunit 239 1926
-4- YKR002W poly(A) polymerase 287 2592
-5- YJR006W DNA polymerase delta 55 kDa subunit 295 2708
-6- YPL167C DNA polymerase zeta subunit 305 2873
-7- YPR010C RNA polymerase I subunit 335 3271
-8- YFL036W mitochondrial RNA polymerase 336 3311
-9- YOL005C RNA polymerase II core subunit 394 3865
-10- YNL262W DNA polymerase II 408 4020
-11- YCL031C involved in rRNA processing 412 4110
-12- YJL140W RNA polymerase II fourth largest subunit 426 4238
-13- YKL144C RNA polymerase III subunit 441 4345
-14- YNL151C HMG1-like protein|RNA polymerase III (C) 31 kDa subunit 465 4532
-15- YDL102W DNA polymerase III catalytic (delta) subunit 471 4578
-16- YDR045C TFIIS-like small Pol III subunit C11 479 4649
-17- YOR151C second largest subunit of RNA polymerase II 481 4687
YIR038C glutathione transferase 0.000863596764119398 183
-1- YIR038C glutathione transferase 57 210
-2- YDR272W glyoxylase-II 86 328
-3- YML004C lactoylglutathione lyase (glyoxalase I) 103 407
-4- YLL060C glutathione transferase 147 922
-5- YOR040W glyoxylase-II 183 1187
total genes measured in network: 744.

This output (`example_result.txt`) lists all the subgraphs that were determined as significantly changed in the analysis. For each subgraph it first gives the "anchor gene", i.e. the local minimum from which the extension was started, and the associated PC-value and maximum rank of subgraph members. This is followed by a list of all member genes of the subgraph, plus their rank among the genes that are present in the evidence network and their rank in the complete gene list, which can be different as not all genes will be annotated in each case – in the present example, only 744 enzymes out of a total of about 6000 genes were analyzed. The significance threshold is determined depending on the total number of genes and the selected sensitivity (specify a higher sensitivity using the option -T, e.g. -T3 admits subgraphs with p-values that are three times higher), the default settings in many cases give a reasonable list of significantly changed groups. For exact statistical confidence assessments, it is possible to analyze random permutations of the data (option -R, see below).

To save the results in a new file, you can specify an additional output filename, e.g.

```
C:\My Documents\GiGA\> GiGA.exe –Iexperimental_data.txt
-Nevidence_network.txt -Ggenenames.txt -Ftxt –Oexample_output.txt
```

The output file will be a tab-delimited text, just like the input files, and can be conveniently analyzed in Excel.

For a more convenient visualization, specify the output format Graph Description Language (`-Fgdl`). The resulting output file can be explored in the graph-layout program aiSee, which is freely available for academic use from http://www.aisee.com (see `example_results.gdl` for the GDL file corresponding to the analysis described above).

In most cases, the maximum size of the determined subgraphs should be restricted to keep visualization simple and to detect significant clusters within subgraphs. The maximum size is set using the option –M, e.g. –M40 limits the size of subgraphs to 40 member genes. The default is 20 genes.

To see the results of a random permutation of the data, specify an additional option (-R). This will randomize the order of genes in the input file. This option should be used to obtain an estimate of the multiple-testing corrected p-values and false-discovery rate for the subgraphs.

## Input file formats

All input files are in tab-delimited format that can be viewed and created in Microsoft Excel. Comments can be included in all files, by putting a # at the beginning of the line.

The following two files are required:

**Microarray results (option -I):** This file should contain a list of gene identifiers in the first column and should be sorted by fold-change or by any other metrics of differential gene expression (e.g. t-statistics). Create separate files for up- and down-regulation, but both should contain *all* the genes that are present on the microarray. It

is also possible to combine the analysis of up- and down-regulated genes in one step, e.g. by sorting the gene list by absolute log ratios. The file may contain additional columns, e.g. the actual expression values. These columns will be ignored by GiGA.

**Network file (option -N):** This file should contain a list of all the edges in the evidence graph. The first two tab-delimited columns in each line specify a gene pair that is connected, the third (optional) column specifies the evidence for that particular link. Multiple specifications of the same edge are ignored. Make sure that the gene identifiers match those in the microarray results file. The file may contain additional columns, e.g. alternative classifications. These columns will be ignored by GiGA.

Alternatively, you can provide a
**Sparse Network file (option –X):** This tab-delimited text file contains a list of nodes (genes) in the evidence graph and the associated evidence. The first column specifies the gene identifier, the second lists all associated evidence terms separated by a vertical line (|). Usually, this file will be much shorter than the standard network file.

The following file is optional:

**Gene names file (option -G):** Contains gene identifiers in the first column and descriptive gene names in the second. Make sure that the gene identifiers match those of the microarray results file. Including gene names makes the results easier to read, but it is not necessary that all genes have a descriptive name.
